# Full Genome Sequence of pT3, a Multiresistant Plasmid Carrying the *mcr-3.5* Colistin Resistance Gene, Recovered from an Extended-Spectrum-β-Lactamase-Producing Escherichia coli Isolate from Crickets Sold as Food

**DOI:** 10.1128/MRA.00647-19

**Published:** 2019-07-18

**Authors:** Katrin Zurfluh, Marc J. A. Stevens, Melissa Bucher, Laurent Poirel, Patrice Nordmann, Roger Stephan

**Affiliations:** a Institute for Food Safety and Hygiene, Vetsuisse Faculty, University of Zurich, Zurich, Switzerland; b Emerging Antibiotic Resistance, Medical and Molecular Microbiology Unit, Department of Medicine, University of Fribourg, Fribourg, Switzerland; c National Reference Center for Emerging Antibiotic Resistance, University of Fribourg, Fribourg, Switzerland; Portland State University

## Abstract

Here, we sequenced multidrug resistance plasmid pT3, which encodes *mcr*-*3.5* and other resistance genes. The cooccurrence of *mcr* genes and other resistance genes on a single plasmid is of concern.

## ANNOUNCEMENT

The last decade has seen a rapid and massive global spread of extended-spectrum-β-lactamase (ESBL)-producing Enterobacteriaceae in many environments, including food (1).

Recently, we isolated an ESBL-producing Escherichia coli strain from crickets of the family Gryllidae that were bought as food in a Thai store in Switzerland. The food sample was homogenized using a stomacher sample blender and incubated at a 1:10 ratio in Enterobacteriaceae enrichment (EE) broth (BD, Franklin Lakes, NJ, USA) at 37°C overnight. For the detection of ESBL producers, chromogenic Brilliance ESBL agar (Oxoid, Hampshire, United Kingdom) was inoculated with one loopful of the enrichment culture. The plate was incubated at 37°C for 24 h under aerobic conditions.

One presumptive-positive E. coli colony on the chromogenic agar was identified by matrix-assisted laser desorption ionization–time of flight mass spectrometry (MALDI-TOF MS) (Biotyper compass explorer software 4.1.60; Bruker Daltonics, Bremen, Germany) and further characterized according to Zurfluh et al. (2).

The strain was characterized as a CTX-M-55-producing Escherichia coli strain belonging to phylogroup A and showing phenotypical resistance to colistin (MIC, 8 mg/liter), ampicillin, cefazolin, cefotaxime, sulfamethoxazole-trimethoprim, kanamycin, gentamicin, tetracycline, and chloramphenicol.

From this strain, plasmids were extracted from a 1-ml overnight culture in brain heart infusion (BHI) broth (Oxoid, Cheshire, England) using the Qiagen midikit (Qiagen, Hombrechtikon, Switzerland) and transferred by transformation using electroporation (3) into E. coli DH5α (Molecular Cloning Laboratories [MCLAB], South San Francisco, CA, USA). Colistin-resistant transformants were selected on LB agar supplemented with 2 mg/liter colistin (Sigma-Aldrich, Buchs SG, Switzerland). From a colistin-resistant transformant, the plasmid pT3 was extracted (250 ng/μl) using a large-construct kit (Qiagen) according to the manufacturer’s protocol. Libraries were prepared using the SMRTbell template prep kit 1.0. according to the manufacturer’s instruction. Sequencing was done using a single PacBio RS II cell (Pacific Biosciences, Menlo Park, CA, USA) and performed at the Functional Genomics Center Zurich (Zurich, Switzerland). The sequencing resulted in 150,292 reads with a total length of 1,738,030,792 bp. Raw reads were assembled using Hierarchical Genome Assembly Process (HGAP) 3.0 in SMRT Portal 3.2 (Pacific Biosciences) using 0.88 as the polymerase read quality cutoff, 100 kb as the genome size, and all other options left unchanged. The polymerase read quality filtering resulted in 1,873 input reads with a total length of 30,356,970 bp. A total of 1,843 of the input reads could be assembled to a single primary contig of 107,601 bp. The primary contig was polished by deleting the repetitive sequence at the contig end, which resulted in a 79,291-bp circular sequence with an approximate coverage of 270-fold and a GC content of 49.7%.

Comparison of pT3 with the plasmid database PlasmidFinder (4) revealed that pT3 belongs to the IncF1A, IncF1B, and IncX1 plasmid incompatibility groups and lacked the genes needed for conjugation. The plasmid was annotated with the NCBI Prokaryotic Genome Annotation Pipeline. It harbors the colistin resistance gene *mcr-3.5*, which is the first time that this gene has been found in Switzerland. Furthermore, pT3 harbors *tet*(A), *bla*_TEM-128_, and *qnrS1* genes, encoding resistance to tetracycline, β-lactams, and quinolones, respectively. In addition, genes encoding resistances to silver, copper, and mercury were located on pT3. Comparison to plasmids in public databases revealed that pT3 is a mosaic plasmid. Mosaic plasmids are composed of genetic elements from distinct sources and are common in Enterobacteriaceae members (5).

A high similarity between pT3 and plasmids from different species was noteworthy, and the insertion of the *mcr-3.5* gene onto such plasmids presents a potential additional threat. The cooccurrence of *mcr* genes and other resistance genes on a single plasmid is of concern.

### Data availability.

The full sequence of pT3 has been deposited in the GenBank database under the accession number MK656937. Reads are available in the Sequence Read Archive (SRA) under study number PRJNA527974, experiment number SRX5905084, and run number SRR9131260.
